# Stabilization of the transcription factors slug and twist by the deubiquitinase dub3 is a key requirement for tumor metastasis

**DOI:** 10.18632/oncotarget.20561

**Published:** 2017-08-24

**Authors:** Yiwei Lin, Yu Wang, Qing Shi, Qian Yu, Cuicui Liu, Jing Feng, Jiong Deng, B. Mark Evers, Binhua P. Zhou, Yadi Wu

**Affiliations:** ^1^ Department of Molecular and Cellular Biochemistry, Lexington, KY, USA; ^2^ Department of Pharmacology & Nutritional Sciences, Lexington, KY, USA; ^3^ Department of Surgery, Lexington, KY, USA; ^4^ Markey Cancer Center, the University of Kentucky, College of Medicine, Lexington, KY, USA; ^5^ Department of Laboratory Medicine & Central Laboratory, Shanghai Fengxian District Central Hospital, Shanghai, China; ^6^ Key Laboratory of Cell Differentiation and Apoptosis of Chinese Minister of Education, Shanghai Jiao Tong University School of Medicine, Shanghai, China

**Keywords:** Dub3, Slug, Twist, IL-6, metastasis

## Abstract

The epithelial-mesenchymal transition (EMT) represents a cellular de-differentiation process that provides cells with the increased plasticity required during embryonic development, tissue remodeling, wound healing and metastasis. Slug and Twist are two key EMT transcription factors (EMT-TFs) that are tightly regulated via ubiquitination and degradation. How Slug and Twist escape degradation and become stabilized in cancer cells remains unclear. One plausible mechanism of Slug and Twist stabilization involves removal of ubiquitin by deubiquitinases (DUBs). In this study, we identified Dub3 as a novel DUB for both Slug and Twist. We further found that Dub3 overexpression increased Slug and Twist protein levels in a dose-dependent manner, whereas Dub3-knockdown decreased their protein levels. Of importance, Dub3 interacted with Slug and Twist and prevented them from degradation, thereby promoting migration, invasion, and cancer stem cell (CSC)-like properties of breast cancer cells. Intriguingly, Dub3 was identified as an early response gene that was upregulated after exposure to inflammatory cytokines such as IL-6, which plays a critical role in the growth and metastasis of breast cancer cells, as well as the maintenance of breast CSCs. We found that Dub3 played an essential role in IL-6 induced EMT through stabilization of Slug and Twist. Our study has uncovered an IL-6-Dub3-Slug/Twist signaling axis during EMT and suggests potential approaches that could target Dub3 to prevent metastatic breast tumor.

## INTRODUCTION

Originally recognized as a vital cellular process during embryonic morphogenesis, EMT has been linked to pathologies including fibrosis and cancer progression, due to the similarities in the migratory and invasive characteristics that the cells obtain during these processes [1-4]. Indeed, growing evidence indicates that EMT endows tumor cells with CSC-like traits, renders them resistant to therapeutics and provides them with proclivity for early metastasis. While EMT is required for dissociation of tumor cells from primary carcinomas and their subsequent migration and dissemination, it is the reversal process mesenchymal-epithelial transition (MET), that directs tumor cells to regain epithelial properties and proliferate at metastatic sites [5, 6]. Not surprisingly, EMT is executed and tightly regulated in response to different levels of signaling integrative of transcriptional control, epigenetic modifications as well as protein stability and subcellular localization [2, 7-15].

Classical EMT-TFs fall into three groups: Snail family zinc finger proteins (Snail/Snail1 and Slug/Snail2); Twist family basic helix-loop-helix (bHLH) proteins (Twist/Twist1 and Twist2); and ZEB family two-handed zinc finger/homeodomain proteins (ZEB1 and ZEB2/SIP-1). Aberrant expression/activation of EMT-TFs can give rise to tumor angiogenesis, invasion and metastasis, confer tumor cells with CSC-like properties as well as resistance to apoptosis. For example, our previous studies along with others’ demonstrated that Snail expression correlates with tumor grade and nodal metastasis of invasive ductal carcinoma [14, 16]; and Slug overexpression prior to neoplastic transformation predisposes BRCA1-mutation carriers to aggressive basal-like breast cancer (BLBC) [17]; and Twist is highly expressed in invasive lobular carcinoma [18]. EMT-TFs are under multiple layers of control. With respect to protein stability, the Snail family members and Twist are extremely labile proteins subject to poly-ubiquitination and degradation mediated by E3 ubiquitin ligases including β-TrCP, Fbxl14, Fbxl5, Fbxo11 and Mdm2 [19].

Ubiquitination is a reversible process, as ubiquitin moieties can be removed from proteins by deubiquitinases (DUBs). DUBs consist of five enzyme families: ubiquitin-specific proteases (USPs), ubiquitin C-terminal hydrolases (UCHs), Machado-Josephin domain proteases (MJDs), ovarian tumor proteases (OTUs) and Jab1/Mov34/MPN^+^ (JAMM) domain-containing metalloenzymes. Recent studies demonstrated that DUBs play essential roles in cancer development through regulation of transcription, DNA repair and cell cycle progression [20]. As such, DUBs have emerged as promising targets of anti-cancer therapies [21]. It is speculative yet interesting to find out whether ubiquitination of Snail family and Twist transcription factors is counteracted by DUBs under specific conditions, such that these transcription factors escape degradation and become stabilized to promote EMT and metastasis. As a member of USPs, Dub3 is an immediate early gene whose transcription can be rapidly induce by cytokines [20, 22, 23]. Importantly, high expression of Dub3 in mouse embryonic stem cells (ESCs) couples the G1/S checkpoint with pluripotency through Cdc25A de-ubiquitination, and Dub3 depletion reduces the proliferative potential of breast cancer cells *in vivo* by degradation of Cdc25A [24, 25].

In the current study, we identified Dub3 as a new DUB of Slug and Twist. We found that Dub3 interacted with and stabilized Slug and Twist through de-ubiquitination. Furthermore, we demonstrated that Dub3 promoted migration, invasion and CSC-like properties of breast cancer cells. Together, our study identifies a novel role of Dub3 in inducing EMT through Slug and Twist stabilization, and strengthens the potential of Dub3 as an anti-cancer drug target.

## RESULTS

### Dub3 stabilizes Slug and Twist

**Figure 1:**
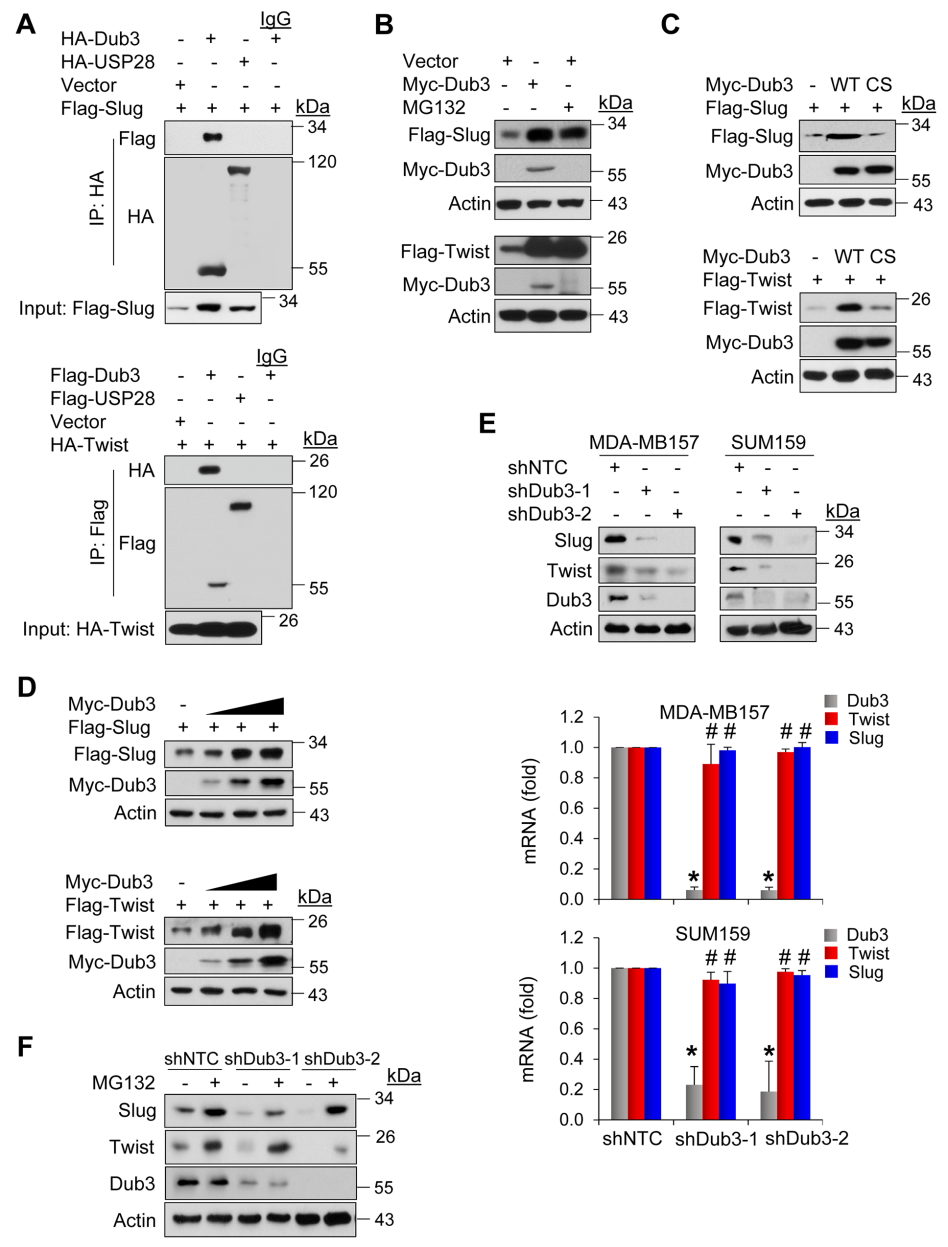
Dub3 stabilized Slug and Twist **(A)** Flag-tagged Slug and HA-tagged DUBs or HA-Twist and Flag-tagged DUBs were co-expressed in HEK293 cells. After immunoprecipitation, bound Slug or Twist was examined by western blot. **(B)** Flag-Slug or Flag-Twist was expressed with or without Myc-Dub3 in HEK293 cells or treated with MG132 only. Expression of Slug, Twist and Dub3 was examined by western blot analysis. **(C)** Flag-Slug or Flag-Twist was co-expressed with wild-type (WT) or C89S (CS) mutant Dub3 in HEK293 cells and examined by western blot analysis. **(D)** Flag-Slug or Flag-Twist was co-expressed with increasing amounts of Myc-Dub3 in HEK293 cells and examined by western blot analysis. **(E)** MDA-MB157 and SUM159 cells were stably transfected with control or Dub3 shRNAs and the expression of Slug, Twist and Dub3 was examined by western blot analysis. The mRNAs of Slug, Twist and Dub3 in MDA-MB157 and SUM159 cells stably transfected with control or Dub3 shRNA were examined by real-time PCR. **(F)** MDA-MB157 cells stably transfected with control or Dub3 shRNA were treated with or without MG132 and expression of Slug, Twist and Dub3 was examined. *p value < 0.001; ^#^p value > 0.05.

To identify specific DUBs involved in mediating protein stability of the Slug, we performed a screening of siRNA library targeting the 99 known or putative DUBs. Based on the initial screening, we identified two potential candidates, USP28 and Dub3 (data not shown). However, we found that only Dub3, but not USP28, interacted with Slug (top panel, Figure 1A). When Dub3 was co-expressed with Slug in HEK293 cells, we found that Dub3 significantly increased protein levels of Slug, an effect comparable to treatment with proteasome inhibitor MG132 (top panel, Figure 1B). Because degradation of both Slug and Twist is mediated by β-TrCP1 and Fbxl14, and DUBs usually counteract E3 ligases to stabilize their target proteins, we asked whether Dub3 also stabilizes Twist. Surprisingly, Dub3 also significantly increased Twist protein (bottom panel, Figure 1B). In contrast, a Dub3 mutant, in which the catalytic cysteine was replaced with serine (C89S, CS), had no effect on Slug/Twist protein levels, indicating that Dub3 enzymatic activity is required for Slug/Twist stabilization (Figure 1C). Similar to Slug, Twist also interacted with Dub3 but not USP28 (bottom panel, Figure 1A). In addition, when we co-expressed Slug/Twist with increasing amounts of Dub3 in HEK293 cells, the levels of Slug and Twist increased in a Dub3 dose-dependent manner (Figure 1D). To test whether endogenous Slug and Twist are subject to similar regulation, we knocked down Dub3 expression in basal-like breast cancer (BLBC) cells MDA-MB157 and SUM159 and found that the protein levels of Slug and Twist, rather than mRNA levels of these two molecules, significantly decreased in these cells (Figure 1E). This excludes the possibility that Dub3 indirectly regulates transcription rather than protein stability of Slug and Twist. Furthermore, MG132 treatment restored the downregulation of Slug and Twist in Dub3-knockdown MDA-MB157 cells (Figure 1F). Collectively, our data indicated that Dub3 is involved in mediating the protein stability of Slug and Twist.

### Dub3 interacts with Slug and Twist

**Figure 2:**
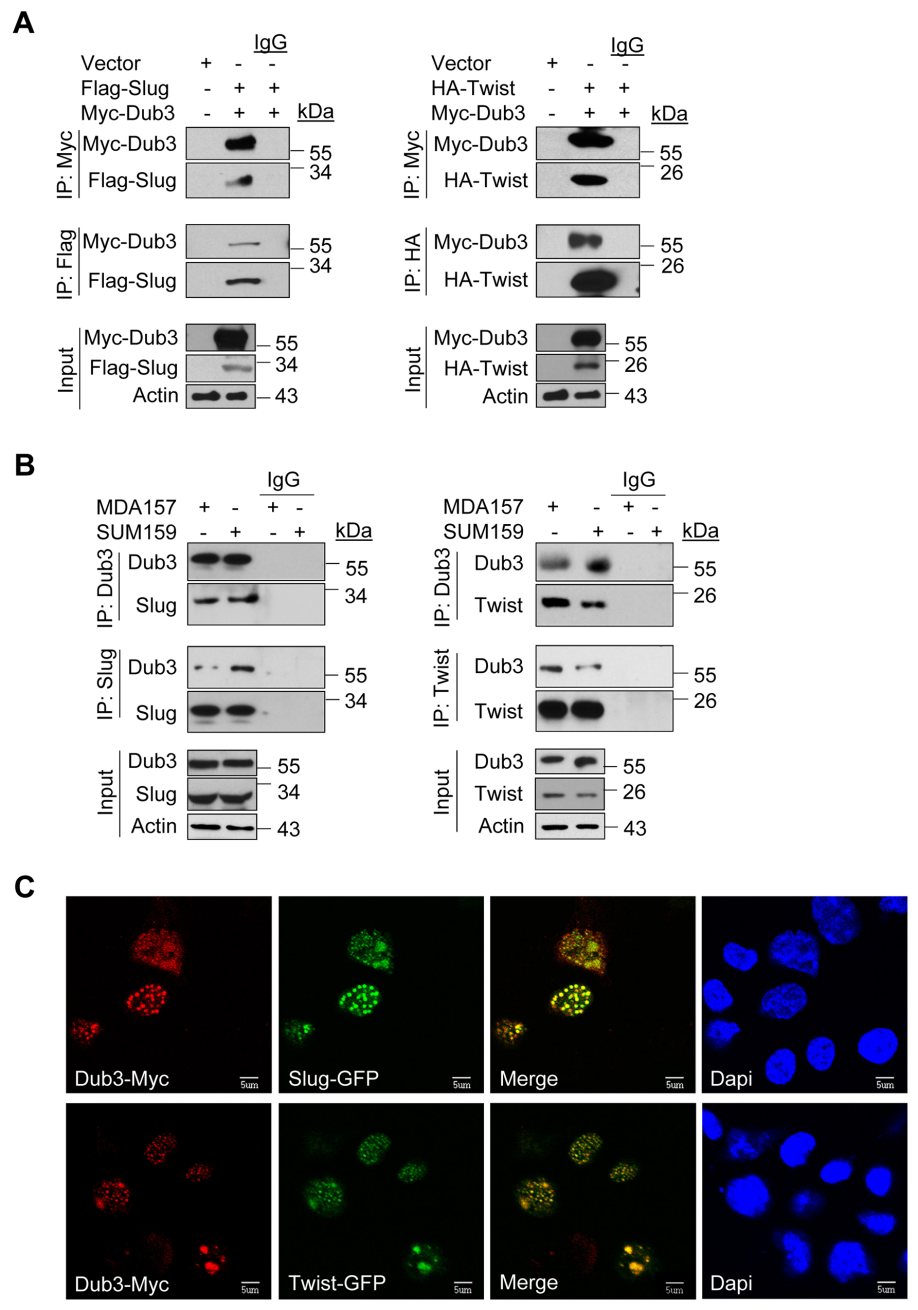
Dub3 interacts with Slug and Twist **(A)** Flag-Slug or HA-Twist was co-expressed with Myc-Dub3 in HEK293 cells. Slug-Dub3 or Twist-Dub3 complexes were immunoprecipitated with either Myc, HA or Flag antibody, and the associated proteins were examined by western blot analysis using HA, Flag and Myc antibody, respectively. **(B)** Endogenous Dub3 and Slug or Twist were immunoprecipitated from MDA-MB157 and SUM159 cells, and the bound endogenous Slug/Twist and Dub3 were examined by western blot analysis. **(C)** EGFP-Slug or EGFP-Twist was co-expressed with Myc-Dub3 in HEK293 cells. After fixation and staining, the cellular location of Slug/Twist (green) and Dub3 (red) was visualized with confocal microscopy. Scale bar = 5 μm.

To further define the mechanism of Dub3-mediated Slug and Twist stabilization, we performed co-immunoprecipitation (Co-IP) experiment using HEK293 cells expressing both Myc-Dub3 and Flag-Slug or HA-Twist. After IP of Dub3, we detected associated Slug/Twist, and vice versa (Figure 2A). Concurrently, we also performed endogenous Co-IP and confirmed the association of Dub3 with Slug and Twist in MDA-MB157 and SUM159 cells (Figure 2B). The Dub3-Slug/Twist interaction was further supported by immunofluorescence (IF) analysis, as Myc-Dub3 co-localized with GFP-Slug or GFP-Twist in the nuclei of HEK293 cells (Figure 2C).

### Dub3 deubiquitinates Slug and Twist

**Figure 3:**
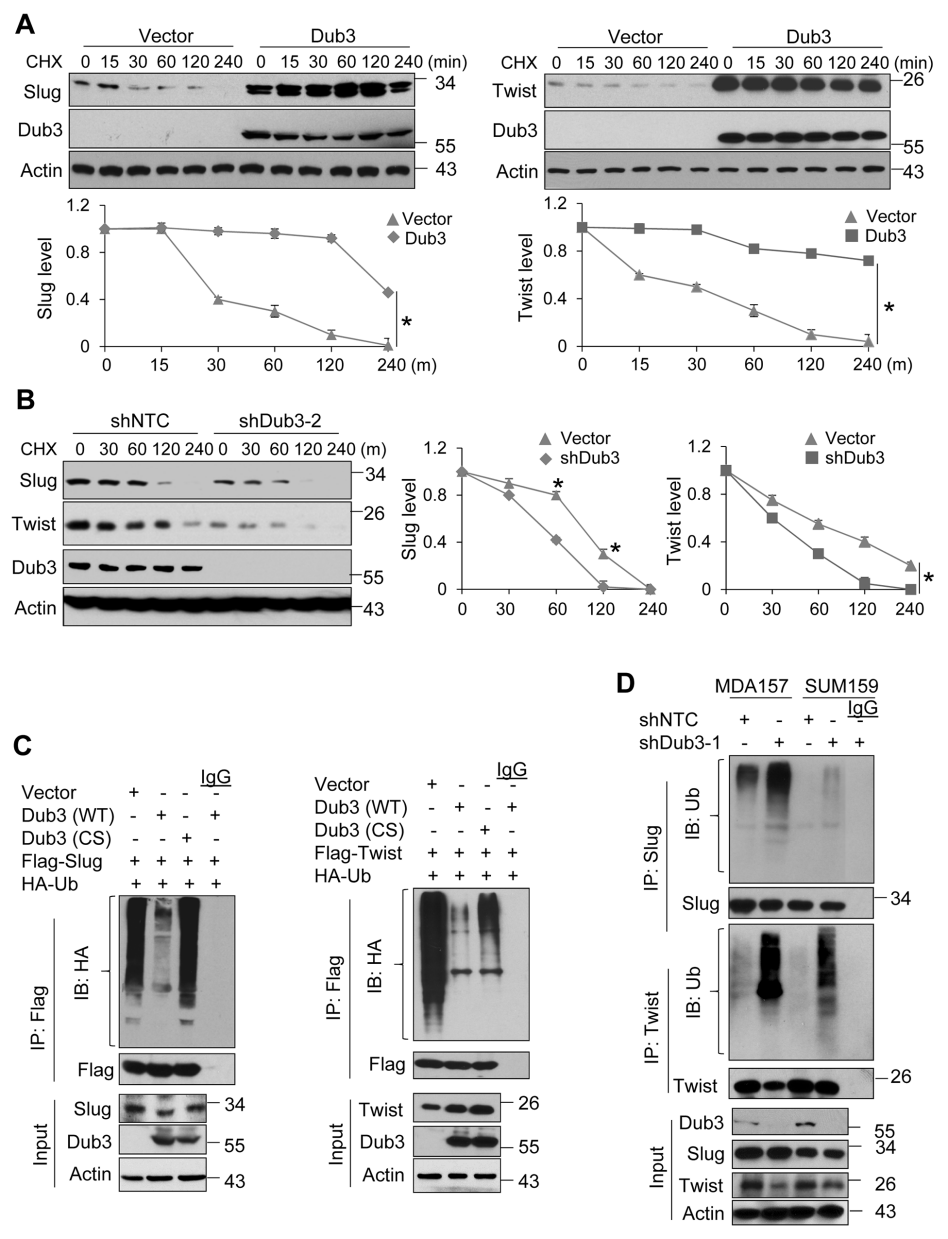
Dub3 de-ubiquitinates Slug and Twist **(A)** Flag-Slug or HA-Twist was co-expressed with Myc-Dub3 or control vector in HEK293 cells. After the cells were treated with cycloheximide (CHX) for indicated time intervals, expression of Slug, Twist and Dub3 was examined by western blot analysis. **(B)** MDA-MB157 cells stably transfected with control or Dub3 shRNA were treated with CHX. Expression of endogenous Slug, Twist and Dub3 was examined. The intensity of Slug/Twist expression for each time point was quantified by densitometry and plotted (bottom panel). The experiment was repeated three times and a representative experiment is presented. *p value < 0.001; **(C)** Flag-Slug or Flag-Twist was co-expressed with HA-ubiquitin and either WT or CS Dub3 in HEK293 cells. After cells were treated with MG132, Slug and Twist proteins were immunoprecipitated and poly-ubiquitination of Slug and Twist detected by western blot analysis using HA antibody. **(D)** MDA-MB157 and SUM159 cells stably transfected with control or Dub3 shRNA were treated with MG132. Cell extracts were immunoprecipitated with Slug or Twist antibody and the poly-ubiquitination of Slug and Twist detected by western blot analysis.

To test the effect of Dub3 on the protein stability of Slug and Twist, we co-expressed Slug/Twist with Dub3 or vector control in HEK293 cells and treated cells with cycloheximide (CHX) at different time intervals to block newly synthesized protein. Both Slug and Twist proteins became significantly stabilized in presence of Dub3 compared to vector control group (Figure 3A). Consistently, CHX chase experiments showed that endogenous Slug and Twist became unstable and degraded rapidly upon knockdown of Dub3 in MDA-MB157 cells (Figure 3B). In Slug or Twist expressing HEK293 cells treated with MG132, IP of Slug/Twist showed that both proteins were heavily ubiquitinated (lane 1 of both upper panels, Figure 3C). Co-expression of wild-type (WT) Dub3 almost completely abolished ubiquitination of Slug and Twist, while co-expression of CS-Dub3 did not show this effect (lane 2 vs lane 3 in both upper panels, Figure 3C). Conversely, the level of Slug and Twist ubiquitination was significantly increased upon Dub3-knockdown in MDA-MB157 and SUM159 cells (Figure 3D). Taken together, our results demonstrated that Slug and Twist associate with Dub3 and are subject to Dub3-mediated de-ubiquitination, which contributes to their protein stabilization.

### Dub3 mediates migration, invasion and CSC-like properties of breast cancer cells

**Figure 4:**
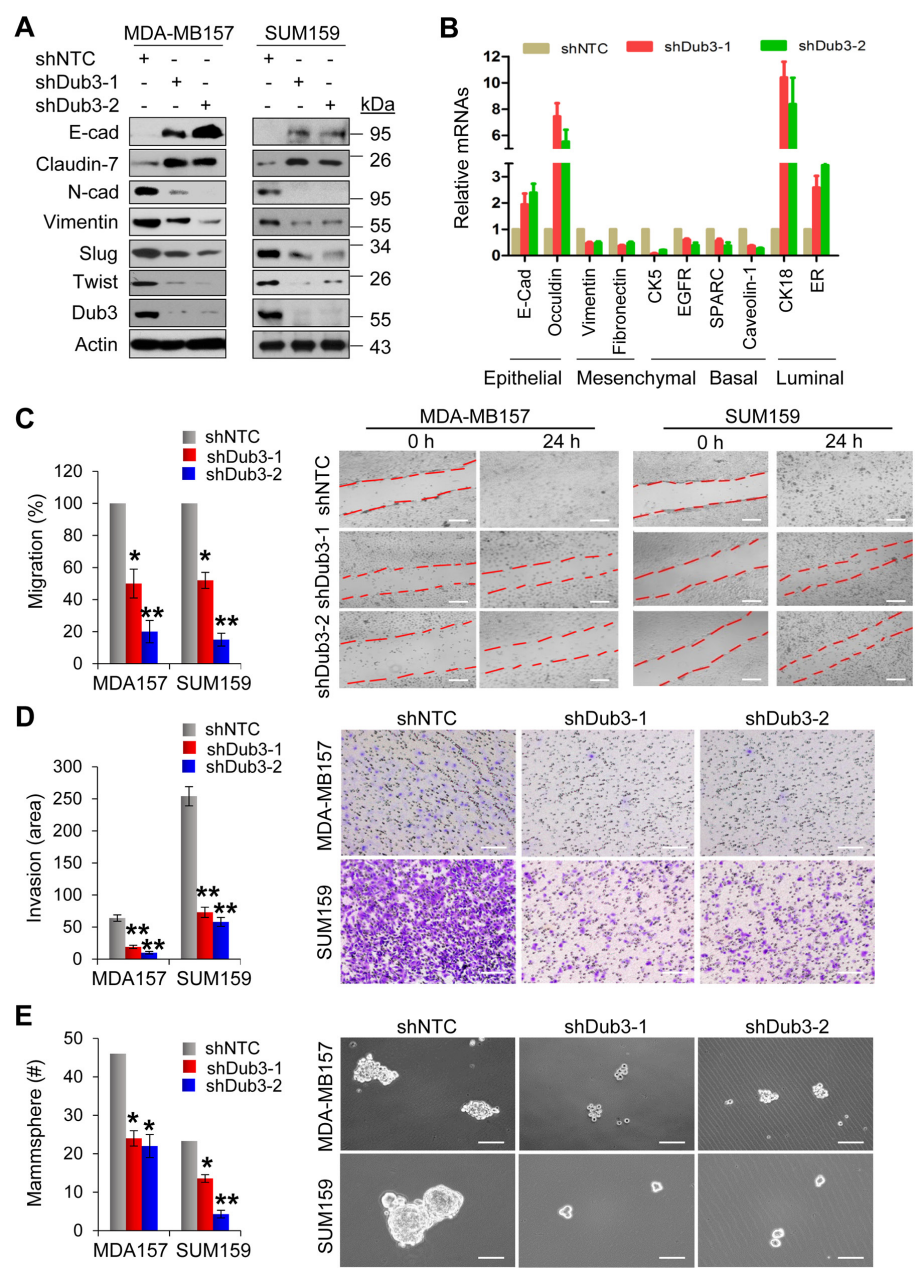
Knockdown of Dub3 inhibits migration, invasion and CSC-like characteristics of BLBC cells **(A)** MDA-MB157 and SUM159 cells stably transfected with control or Dub3 shRNA were examined by western blot analysis for expression of various protein markers as indicated. **(B)** The mRNA levels of various markers as indicated were quantitated by real-time PCR (mean ± SD in three separate experiments). **(C)** Wound healing assay of MDA-MB157 and SUM159 cells with or without stable knockdown of Dub3. Data were presented as mean ± SEM. **(D)** Boyden chamber invasion assay of MDA-MB157 and SUM159 cells with or without stable knockdown of Dub3. Data were presented as mean ± SEM. **(E)** Quantification of tumorsphere from MDA-MB157 and SUM159 cells stably expressing control or Dub3 shRNA (mean ± SD from three independent experiments) (left panel). Representative images were shown (right panel). Scale bar = 100 μm. **p value < 0.01; *p value <0.05.

To investigate the function of Dub3 in breast cancer, we established stable MDA-MB157 and SUM159 cells with knockdown of Dub3 expression. Dub3-knockdown increased the expression of epithelial markers E-cadherin and Claudin-7, while down-regulating mesenchymal molecules N-cadherin and Vimentin (Figure 4A). In addition, both cells with Dub3-knockdown showed reduced levels of molecules associated with BLBC such as CK5, EGFR, SPARC and Caveolin-1, and gained expression of luminal markers such as CK18 and ERα (Figure 4B). We then performed migration and invasion assays and found that Dub3-knockdown significantly decreased migratory and invasive capabilities of MDA-MB157 and SUM159 cells (Figure 4C-4D). Furthermore, to assess the effect of Dub3 in regulating CSC-like properties, we performed tumorsphere-formation assays and found that Dub3-knockdown dramatically reduced the number and size of tumorspheres in MDA-MB157 and SUM159 cells (Figure 4E).

**Figure 5:**
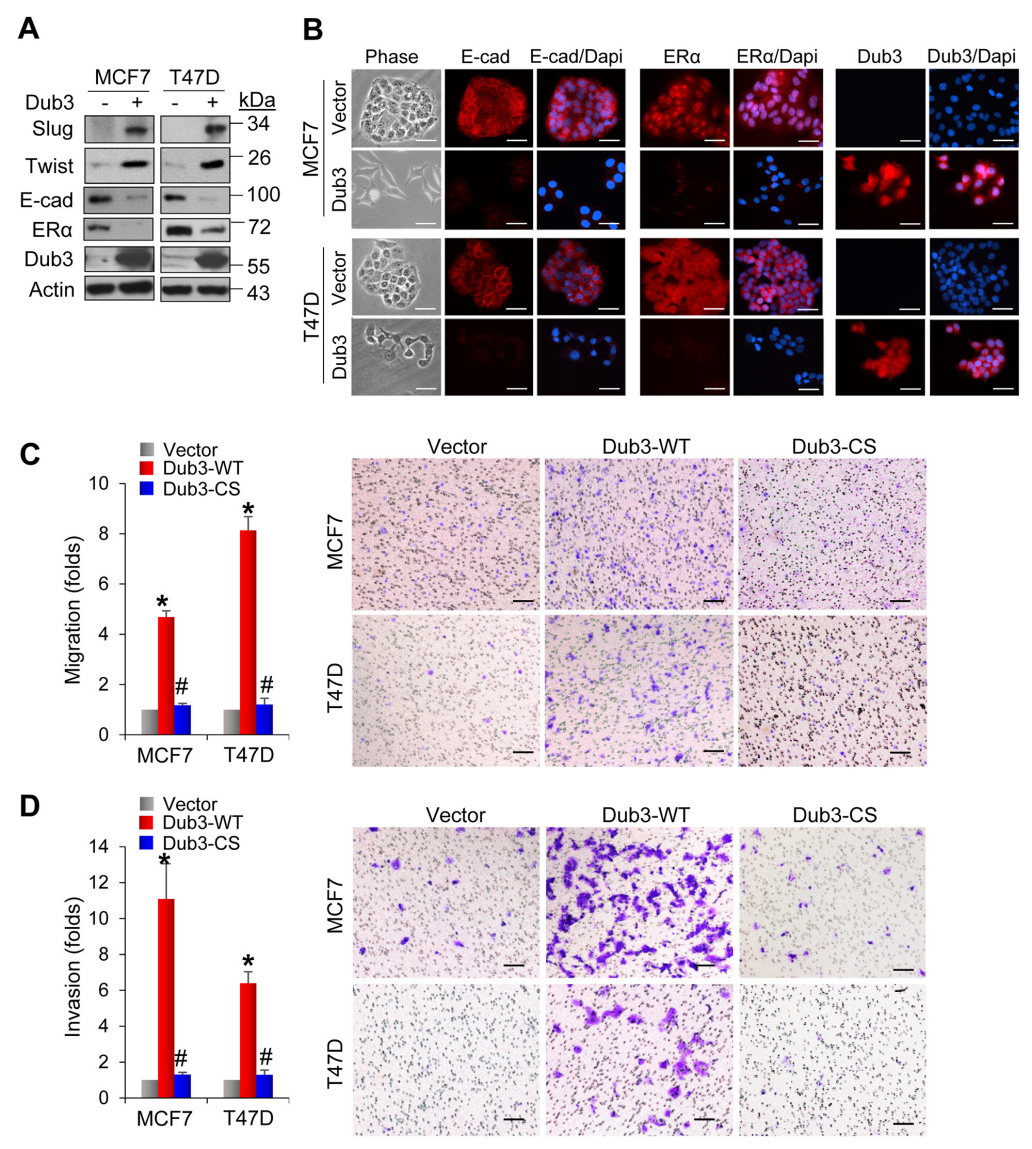
Overexpression of Dub3 induces EMT **(A)** Dub3 was stably transduced in MCF7 and T47D cells and the indicated proteins were examined by western blot analysis. **(B)** MCF7 and T47D cells with Dub3 overexpression were examined for morphologic changes as well as expression of E-cadherin, ER α and Dub3 with IF. **(C)** Boyden chamber migration assay of MCF7 and T47D cells with Dub3 WT or CS mutant expression. Data were presented as mean ± SEM. **(D)** Boyden chamber invasion assay of MCF7 and T47D cells with Dub3 WT or CS mutant expression. Data were presented as mean ± SEM. Scale bar = 100 μm. *p value < 0.001; ^#^p value > 0.05.

To further investigate the roles of Dub3, we examined whether Dub3 expression can confer luminal breast cancer cells with EMT phenotypes. MCF7 and T47D cells are typical luminal cells with little endogenous Dub3 expression. Expression of Dub3 in these two cell lines dramatically increased the levels of Slug and Twist (Figure 5A) and reduced E-cadherin and ER expression. Concomitantly, Dub3 expression induced significant morphology changes as cells turned into a scattered appearance indicative of EMT (Figure 5B). As expected, Dub3 expression dramatically enhanced the migratory and invasive capabilities of both MCF7 and T47D cells (Figure 5C-5D). However, CS-Dub3 lost the ability to increase cell migration and invasion in these cells. Together these results indicated that Dub3 is a critical EMT-promoting factor that can increase migration, invasion and CSC-like properties of breast cancer cells.

### Dub3 is critical for IL-6-induced EMT

**Figure 6:**
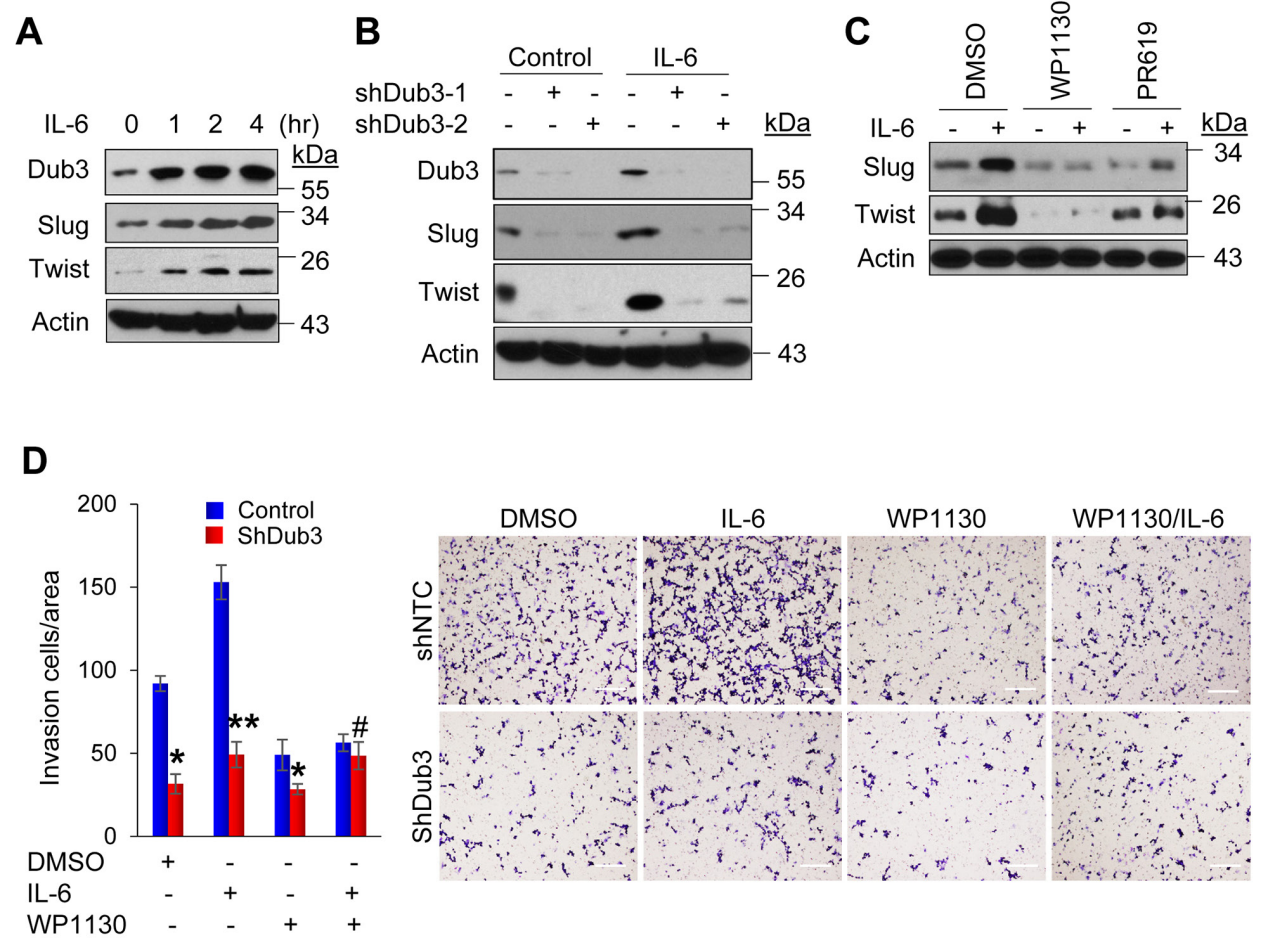
IL-6-induced BLBC cells invasion is mediated by Dub3 **(A)** SUM159 cells were starved overnight and treated with IL-6 for the indicated time intervals and the expression of Dub3, Slug and Twist were examined by western blot analysis. **(B)** SUM159 cells stably transduced with either control or Dub3 shRNA were starved and treated with IL-6 and the expression of Slug and Twist was examined. **(C)** SUM159 cells were pre-treated with DMSO, WP1130 or PR619 for half hour. The cells were then treated with IL-6 (50ng/ml) and the expression of Slug and Twist was examined by western blot analysis. **(D)** SUM159 cells stably transduced with control or Dub3 shRNA were pre-treated with DMSO or WP1130 for half hour. Boyden chamber invasion assay was performed with or without IL-6 induction. Data were presented as mean ± SEM. Scale bar = 100 μm. **p value < 0.01; *p value <0.05; ^#^p value > 0.05.

Dub3 was identified as an early response gene that can be induced by IL-6 [22, 23]. Importantly, IL-6 can induce EMT through the STAT3 signaling, and its level increases with higher breast tumor grade and correlates with metastasis and poor patient survival [26, 27]. To test whether IL-6 promotes EMT through induction of Dub3 and subsequently results in Slug and Twist stabilization, we treated SUM159 cells with IL-6 at different time intervals. Consistent with previous findings, Dub3 was rapidly induced after one hour of IL-6 stimulation, which was accompanied by an increase of Slug and Twist protein levels (Figure 6A). In contrast, Dub3-knockdown almost completely abolished IL-6 induced Slug and Twist upregulation in SUM159 cells (Figure 6B). We also found that a Dub3 inhibitor, WP1130, can dramatically suppress Slug and Twist upregulation induced by IL-6 while PR619 (a deubiquitinating enzyme inhibitor) was less effective in SUM159 cells (Figure 6C) [28, 29]. Furthermore, Dub3-knockdown or WP1130 treatment significantly inhibited IL-6-mediated invasion of tumor cells (Figure 6D); however, WP1130 could not suppress IL-6-induced invasion in Dub3-knockdown cells. Together, these data support the idea that Dub3 functions as a molecule sensor, which can stabilize Slug/Twist and induce EMT in responding to microenvironmental IL-6 stimulation.

### Dub3, Slug and Twist are coordinately expressed in breast tumors

**Figure 7:**
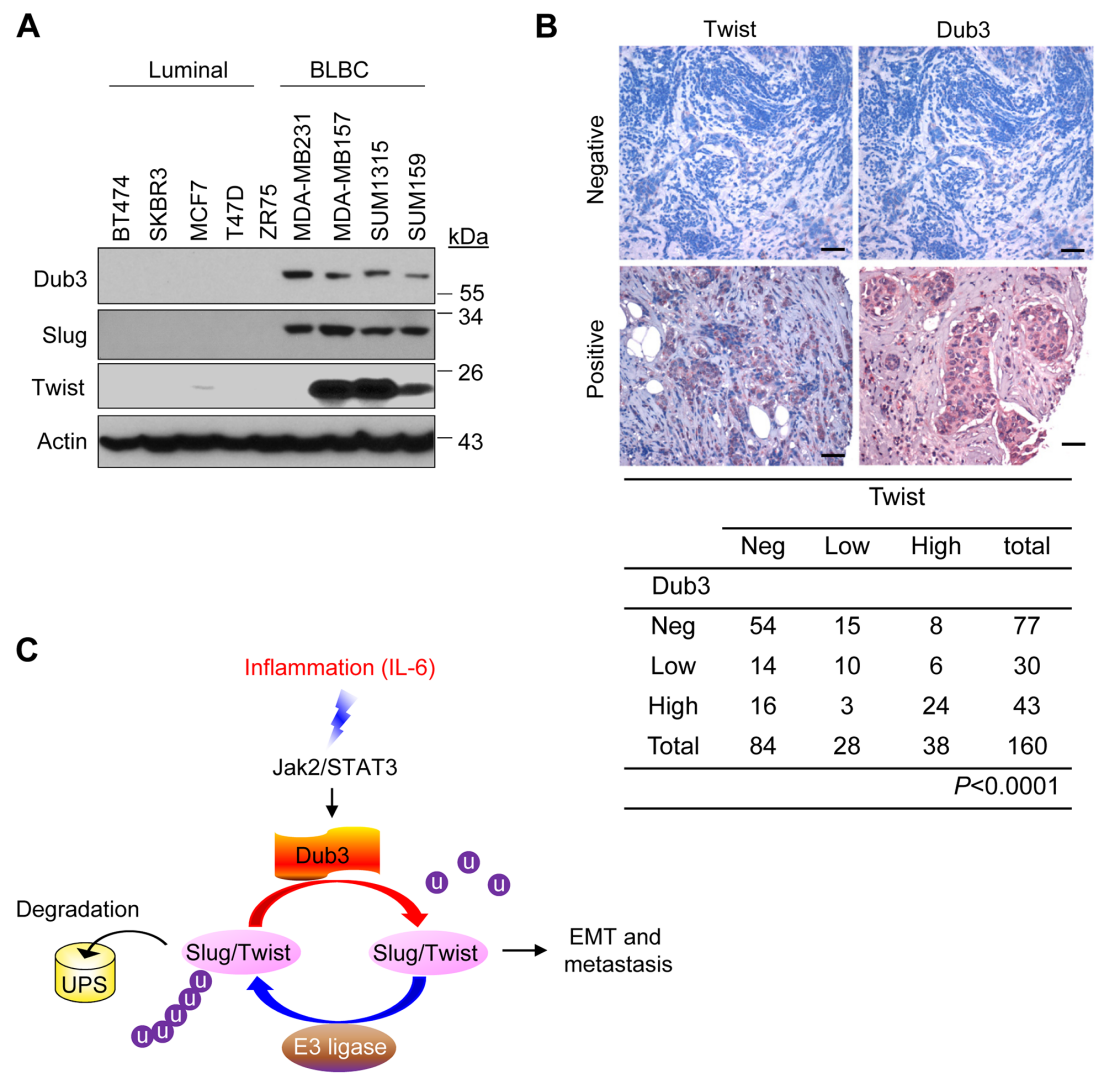
Dub3, Slug and Twist are coordinately expressed in breast tumors **(A)** Expression of Dub3, Slug and Twist was examined in multiple breast cancer cell lines. **(B)** Breast tumor surgical specimens were immunostained using antibodies against Dub3 and Twist. Representative images of IHC staining from the same tumor samples were shown in the top panel and statistical analysis shown in the bottom panel. **(C)** The proposed model of the counteracting effect of Dub3 and E3 ligases of Slug and Twist in mediating EMT and metastasis. Scale bar = 100 μm.

To find out whether the expression of Dub3, Slug and Twist are correlated in breast cancer, we examined their expression in multiple breast cancer cell lines and tumor specimens. As expected, Dub3, Slug and Twist levels are coordinately high in BLBC cell lines compared to luminal breast cancer cell lines (Figure 7A). We also performed immunohistochemistry (IHC) analysis to examine their expression in primary breast tumor specimens. Although we could not detect Slug expression in these tumor samples due to the lack of specific antibodies, we found a significant correlation of Dub3 and Twist in terms of protein intensity and distribution in these breast tumor specimens (Figure 7B). The tumor-staining data support our findings in cell-culture experiments and further strengthen our notion that Dub3 is a critical deubiquitinase in stabilizing Slug and Twist in breast cancer.

## DISCUSSION

Metastasis remains a daunting threat, as approximately 90% of cancer deaths are caused by the spread of tumor cells to other organs [30]. BLBC carries the poorest prognosis among breast cancer subtypes, given its tendency toward early and recurrent metastasis and lack of effective targeted therapies [31-35]. BLBC intrinsically activates EMT in response to various signaling from microenvironment, particularly those from inflammatory cytokines. Similar to many transcription factors, such as p53 and HIF1α, EMT-TFs like Snail, Slug and Twist are often subjected to ubiquitination/degradation and have very short half-lives. Several ubiquitin ligases have so far been linked to the degradation of EMT-TFs. For example, β-TrCP1 has been shown to mediate cytoplasmic degradation of Snail [14, 36]. To be targeted by ubiquitin ligase machinery, a protein usually contains a degradation signal or degron. As for Snail, the degron lies in the β-TrCP1 destruction box DSGXXS at the central region, with the two serine residues phosphorylated by GSK-3β [14]. The lysine residues modified by β-TrCP1 have also been identified as K98, K137 and K146 [37]. Interestingly, Slug and Twist are also subject to ubiquitination and degradation mediated by β-TrCP1, although the classical destruction motif is absent [17, 38]. In addition to β-TrCP1, Fbxl14 is considered a common regulator of Snail/Slug, Twist and ZEB family members [37, 39, 40]. Slug and Twist interact with Fbxl14 through the C-terminal WR domain and an N-terminal hydrophobic sequence, respectively [39, 40]. Furthermore, Fbxl5 exerts its E3 ligase function specifically on Snail and Slug [41]. In spite of these findings, little was known about how the reversal de-ubiquitination process might rescue these EMT-TFs from the fate of degradation during EMT and metastasis. Recently, we and others have identified Dub3 functions as a Snail deubiquitinase and blocks the activity of β-TrCP1 and Fbxl14 to stabilize Snail [29, 42]; however, it remains unclear whether Dub3 can also remove the ubiquitination of Slug and Twist, given that these EMT-TFs share a common ubiquitin E3 ligase β-TrCP1 and Fbxl14. Our current results demonstrated that Dub3 interacts with Slug and Twist and prevents Slug and Twist from degradation mediated by these E3 ligases. The “Ying-Yang” control model proposed here greatly strengthens the concept of cellular plasticity and heterogeneity in EMT (Figure 7C). It mirrors the prominent idea of “partial EMT”, which centers on cells hovering between epithelial and mesenchymal states as they migrate, therefore retaining greater flexibility to switch back through MET to fulfil metastatic colonization. In this regard, the balance between degradation and stabilization of EMT-TFs may oscillate under extremely dynamic cellular contexts, which warrants further investigation.

Furthermore, we found that Dub3 is responsible for Slug and Twist stabilization and EMT induction in BLBC cells, and its expression positively correlates with Twist level in human breast tumor samples. In addition, Dub3 can be rapidly induced by inflammatory cytokine IL-6 [22, 23]. Elevated IL-6 has been shown to confer cancer cells with chemotherapy resistance and contribute to tumor recurrence and metastasis [26, 27]. As IL-6 is highly expressed in BLBC cells as well as infiltrated tumor-associated macrophages (TAMs) [43], it may exert its function of Dub3 activation through autocrine and paracrine loops. Interestingly, IL-6 can stimulate casein kinase 2 (CK2)-mediated phosphorylation and stabilization of Twist in head and neck cancer cells [44]. In the current study, we identified a novel connection between IL-6 and Slug/Twist stabilization in BLBC cells specifically mediated by Dub3. In this regard, Dub3 may represent an excellent example of a molecular sensor that relays environmental signals to intracellular EMT-TFs for the induction of EMT. Interestingly, tumor microenvironment produces cytokines, chemokines and growth factors, including TNFα, TGFβ, IL-6, FGF, epidermal growth factor (EGF), and HGF. These factors initiate and propel tumor metastasis through induction of EMT [45]. Here we have demonstrated that IL-6 promotes metastasis through Dub3 induction. Dub3 can also be induced in response to IL-4 stimulation [23, 46]. Whether other cytokines and/or growth factors may also induce tumor invasion and metastasis via Dub3 is an important area that requires further investigations.

Finally, the discovery of the role of Dub3 in controlling EMT-TFs brings out new therapeutic indications. Development of Slug and Twist-targeting inhibitors would encounter formidable obstacles due to their lack of ligand-binding domains. Our study propels alternative approaches, i.e., design of specific Dub3 enzymatic inhibitors or Dub3-Slug/Twist protein complex-disrupting molecules. Indeed, we found that application of WP1130, a Dub3 inhibitor, not only significantly suppressed Slug and Twist upregulation induced by IL-6 in BLBC cells, but also inhibited the IL-6-induced cell invasion. Our study highlights the perspectives of small molecule compounds that target the IL-6-Dub3-Slug/Twist signaling axis with high specificity and efficiency in treatment of tumor metastasis.

## MATERIALS AND METHODS

### Plasmids and reagents

Both HA-tagged and Flag-tagged full length Twist and Slug were cloned into pcDNA3.0. In addition, full length Twist and Slug were cloned into pEGFP-N1. The WT Dub3 construct was purchased from Addgene. CS Dub3 was generated using the QuikChange Mutagenesis kit (Stratagene, La Jolla, CA, USA) as described previously [47]. All sequences were verified by DNA sequencing. The antibodies used include: anti-Flag, anti-Actin, anti-Myc (Sigma-Aldrich, St. Louis, MO, USA), anti-Dub3 (Abcam), anti-Ub (Millipore), N-cadherin (Upstate), anti-Slug, anti-Twist (Cell Signaling), anti-Vimentin, anti-ERα (Neomarkers), anti-HA (Roche), anti-E-cadherin, anti-Claudin-7 (BD Bioscience). Dub3 shRNA expression plasmids were purchased from MISSION shRNA at Sigma-Aldrich (St. Louis, MO, USA). WP1130 and PR619 were from Selleck. Smartpool siRNA against human Dub3 was from Dharmacon (Chicago, IL, USA).

### Cell culture

The human embryonic kidney HEK293, and breast cancer cell lines, MDA-MB157, MCF7 were purchased from the American Type Culture Collection (Manassas, VA, USA) and grown in Dulbecco’s modified Eagle’s/F12 medium plus 10% fetal bovine serum as described previously. Breast cancer cell lines T47D were grown in RPMI1640 plus 10% FBS. The culture medium for SUM159 is Ham’s F-12 (Invitrogen) supplemented with 5% FBS, 5 μg/mL insulin, and 1 μg/mL hydrocortisone (both from Sigma, St. Louis, MO, USA).

### Immunoprecipitation and western blot analysis

For protein extraction, 5 × 10^5^ cells/well were plated onto six-well plates and transiently transfected with indicated expression plasmids. 48 h after transfection, cells were incubated with or without the proteasome inhibitor MG132 (10 μM) for an additional 6 h before protein extraction and western blot analysis. Primary antibodies against Flag (M2, 1:1000) and HA (3F10, 1:4000) were used for protein detection. For immunoprecipitation (IP), cells were lysed in IP buffer (50 mM Tris (pH 7.5), 150 mM NaCl, 5 μg/ml aprotinin, 1 μg/ml pepstatin, 1% NP-40, 1 mM EDTA and 0.25% deoxycholate). Pre-cleared cell lysates were incubated overnight with 1 μg of anti-HA or anti-Flag antibody conjugated to agarose beads (Roche Molecular Biochemicals) at 4°C. The beads were then washed with PBS, and the immunoprecipitated protein complexes were resolved by 10% SDS–PAGE.

### *In vivo* ubiquitination assay

HEK293 cells were transfected with HA-ubiquitin, Myc-Dub3 and Flag-Twist or Flag-Slug plasmids as indicated. After 48 hours of transfection the cells were treated with 10 μM MG132 for 6 hours. The cells lysates were immunoprecipitated using anti-Flag agarose (Sigma).

### Immunofluorescence staining

For immunofluorescence microscopy, cells transfected with Myc-Dub3 and EGFP-Twist or EGFP-Slug were grown on cover slips, fixed with 4% paraformaldehyde and incubated overnight with anti-Myc antibody. Proteins were visualized by incubation with goat anti-mouse conjugated with Alexa Fluor 568 (Invitrogen, Carlsbad, CA, USA). Finally, cells on cover slips were incubated with 4′, 6′-diamidino-2-phenylindole (Sigma-Aldrich) for 20 min and visualized with a confocal microscope.

### Quantitative real-time PCR

Total RNA was isolated using RNeasy Mini kit (Qiagen) according to the manufacturer’s instructions. Specific quantitative real-time PCR experiments were performed using SYBR Green Power Master Mix following manufacturer’s protocol (Applied Biosystems).

### Invasion assay

Invasion assays were performed in Boyden chambers coated with Matrigel (BD biosciences, San Jose, CA, USA). Cancer cells were seeded on top of the Matrigel in the upper chamber while the bottom chambers were filled with non-serum culture medium plus 100 nM Lysophosphatidic acid (LPA). The invasive cancer cells were stained with crystal violent. All experiments were performed in triplicate.

### Tumorsphere-formation assay

Tumorsphere cultures were performed as described in Dontu et al [48]. In brief, Cell monolayers were plated as single-cell suspensions on ultra-low attachment plates (Corning) in DMEM/F12 medium supplemented with 20 ng/ml EGF, 10 μg/ml insulin, 0.5 μg/ml hydrocortisone and B27. Tumorspheres were counted via visual inspection after 5-10 days.

### Immunohistochemical (IHC) staining

Breast cancer TMA was purchased from US Biomax and Pantomics containing 160 cases of breast cancer. Tissue samples were stained with anti-Dub3 (Abcam) and anti-Twist antibodies (BD company) and each sample was scored by an H-score method that combines the values of immunoreaction intensity and the percentage of tumor cell staining as described previously [49]. In brief, a composite staining index is the product staining intensity (range 0-3) and staining percentage of tumor cell sin each core. The results were ranked in three expression levels: high (3+), Low (2+), and negative (0-1+). Chi-square analysis was used to analyze the relationship between Dub3 and Twist expression; statistical significance was defined as *P*<0.05.

## References

[1] Nieto MA (2002). The snail superfamily of zinc-finger transcription factors. Nat Rev Mol Cell Biol.

[2] Peinado H, Olmeda D, Cano A (2007). Snail, Zeb and bHLH factors in tumour progression: an alliance against the epithelial phenotype?. Nat Rev Cancer.

[3] Thiery JP, Sleeman JP (2006). Complex networks orchestrate epithelial-mesenchymal transitions. Nat Rev Mol Cell Biol.

[4] Thiery JP, Acloque H, Huang RY, Nieto MA (2009). Epithelial-mesenchymal transitions in development and disease. Cell.

[5] Alderton GK (2012). Metastasis: epithelial to mesenchymal and back again. Nat Rev Cancer.

[6] Brabletz T (2012). EMT and MET in metastasis: where are the cancer stem cells?. Cancer Cell.

[7] Lamouille S, Xu J, Derynck R (2014). Molecular mechanisms of epithelial-mesenchymal transition. Nat Rev Mol Cell Biol.

[8] Lamouille S, Subramanyam D, Blelloch R, Derynck R (2013). Regulation of epithelial-mesenchymal and mesenchymal-epithelial transitions by microRNAs. Curr Opin Cell Biol.

[9] Warzecha CC, Carstens RP (2012). Complex changes in alternative pre-mRNA splicing play a central role in the epithelial-to-mesenchymal transition (EMT). Semin Cancer Biol.

[10] Díaz-López A, Moreno-Bueno G, Cano A (2014). Role of microRNA in epithelial to mesenchymal transition and metastasis and clinical perspectives. Cancer Manag Res.

[11] Lin Y, Dong C, Zhou BP (2014). Epigenetic regulation of EMT: the Snail story. Curr Pharm Des.

[12] Lin Y, Wu Y, Li J, Dong C, Ye X, Chi YI, Evers BM, Zhou BP (2010). The SNAG domain of Snail1 functions as a molecular hook for recruiting lysine-specific demethylase 1. EMBO J.

[13] Shi J, Wang Y, Zeng L, Wu Y, Deng J, Zhang Q, Lin Y, Li J, Kang T, Tao M, Rusinova E, Zhang G, Wang C (2014). Disrupting the interaction of BRD4 with diacetylated Twist suppresses tumorigenesis in basal-like breast cancer. Cancer Cell.

[14] Zhou BP, Deng J, Xia W, Xu J, Li YM, Gunduz M, Hung MC (2004). Dual regulation of Snail by GSK-3beta-mediated phosphorylation in control of epithelial-mesenchymal transition. Nat Cell Biol.

[15] Lin Y, Kang T, Zhou BP (2014). Doxorubicin enhances Snail/LSD1-mediated PTEN suppression in a PARP1-dependent manner. Cell Cycle.

[16] Blanco MJ, Moreno-Bueno G, Sarrio D, Locascio A, Cano A, Palacios J, Nieto MA (2002). Correlation of Snail expression with histological grade and lymph node status in breast carcinomas. Oncogene.

[17] Wu ZQ, Li XY, Hu CY, Ford M, Kleer CG, Weiss SJ (2012). Canonical Wnt signaling regulates Slug activity and links epithelial-mesenchymal transition with epigenetic Breast Cancer 1, Early Onset (BRCA1) repression. Proc Natl Acad Sci USA.

[18] Yang J, Mani SA, Donaher JL, Ramaswamy S, Itzykson RA, Come C, Savagner P, Gitelman I, Richardson A, Weinberg RA (2004). Twist, a master regulator of morphogenesis, plays an essential role in tumor metastasis. Cell.

[19] Díaz VM, Viñas-Castells R, García de Herreros A (2014). Regulation of the protein stability of EMT transcription factors. Cell Adhes Migr.

[20] Reyes-Turcu FE, Ventii KH, Wilkinson KD (2009). Regulation and cellular roles of ubiquitin-specific deubiquitinating enzymes. Annu Rev Biochem.

[21] Chauhan D, Tian Z, Nicholson B, Kumar KG, Zhou B, Carrasco R, McDermott JL, Leach CA, Fulcinniti M, Kodrasov MP, Weinstock J, Kingsbury WD, Hideshima T (2012). A small molecule inhibitor of ubiquitin-specific protease-7 induces apoptosis in multiple myeloma cells and overcomes bortezomib resistance. Cancer Cell.

[22] Baek KH (2006). Cytokine-regulated protein degradation by the ubiquitination system. Curr Protein Pept Sci.

[23] Burrows JF, McGrattan MJ, Rascle A, Humbert M, Baek KH, Johnston JA (2004). DUB-3, a cytokineinducible deubiquitinating enzyme that blocks proliferation. J Biol Chem.

[24] Pereg Y, Liu BY, O’Rourke KM, Sagolla M, Dey A, Komuves L, French DM, Dixit VM (2010). Ubiquitin hydrolase Dub3 promotes oncogenic transformation by stabilizing Cdc25A. Nat Cell Biol.

[25] van der Laan S, Tsanov N, Crozet C, Maiorano D (2013). High Dub3 expression in mouse ESCs couples the G1/S checkpoint to pluripotency. Mol Cell.

[26] Sullivan NJ, Sasser AK, Axel AE, Vesuna F, Raman V, Ramirez N, Oberyszyn TM, Hall BM (2009). Interleukin-6 induces an epithelial-mesenchymal transition phenotype in human breast cancer cells. Oncogene.

[27] Berishaj M, Gao SP, Ahmed S, Leslie K, Al-Ahmadie H, Gerald WL, Bornmann W, Bromberg JF (2007). Stat3 is tyrosine-phosphorylated through the interleukin-6/glycoprotein 130/Janus kinase pathway in breast cancer. Breast Cancer Res.

[28] Altun M, Kramer HB, Willems LI, McDermott JL, Leach CA, Goldenberg SJ, Kumar KG, Konietzny R, Fischer R, Kogan E, Mackeen MM, McGouran J, Khoronenkova SV (2011). Activity-based chemical proteomics accelerates inhibitor development for deubiquitylating enzymes. Chem Biol.

[29] Wu Y, Wang Y, Lin Y, Liu Y, Wang Y, Jia J, Singh P, Chi YI, Wang C, Dong C, Li W, Tao M, Napier D (2017). Dub3 inhibition suppresses breast cancer invasion and metastasis by promoting Snail1 degradation. Nat Commun.

[30] Van’t Veer LJ, Weigelt B (2003). Road map to metastasis. Nat Med.

[31] Badve S, Dabbs DJ, Schnitt SJ, Baehner FL, Decker T, Eusebi V, Fox SB, Ichihara S, Jacquemier J, Lakhani SR, Palacios J, Rakha EA, Richardson AL (2011). Basal-like and triplenegative breast cancers: a critical review with an emphasis on the implications for pathologists and oncologists. Mod Pathol.

[32] Fadare O, Tavassoli FA (2008). Clinical and pathologic aspects of basal-like breast cancers. Nat Clin Pract Oncol.

[33] Korsching E, Jeffrey SS, Meinerz W, Decker T, Boecker W, Buerger H (2008). Basal carcinoma of the breast revisited: an old entity with new interpretations. J Clin Pathol.

[34] Kreike B, van Kouwenhove M, Horlings H, Weigelt B, Peterse H, Bartelink H, van de Vijver MJ (2007). Gene expression profiling and histopathological characterization of triple-negative/basallike breast carcinomas. Breast Cancer Res.

[35] Rakha EA, Reis-Filho JS, Ellis IO (2008). Basal-like breast cancer: a critical review. J Clin Oncol.

[36] Domínguez D, Montserrat-Sentís B, Virgós-Soler A, Guaita S, Grueso J, Porta M, Puig I, Baulida J, Francí C, García de Herreros A (2003). Phosphorylation regulates the subcellular location and activity of the snail transcriptional repressor. Mol Cell Biol.

[37] Viñas-Castells R, Beltran M, Valls G, Gómez I, García JM, Montserrat-Sentís B, Baulida J, Bonilla F, de Herreros AG, Díaz VM (2010). The hypoxia-controlled FBXL14 ubiquitin ligase targets SNAIL1 for proteasome degradation. J Biol Chem.

[38] Kim JY, Kim YM, Yang CH, Cho SK, Lee JW, Cho M (2012). Functional regulation of Slug/Snail2 is dependent on GSK-3β-mediated phosphorylation. FEBS J.

[39] Vernon AE, LaBonne C (2006). Slug stability is dynamically regulated during neural crest development by the F-box protein Ppa. Development.

[40] Lander R, Nordin K, LaBonne C (2011). The F-box protein Ppa is a common regulator of core EMT factors Twist, Snail, Slug, and Sip1. J Cell Biol.

[41] Viñas-Castells R, Frías Á, Robles-Lanuza E, Zhang K, Longmore GD, García de Herreros A, Díaz VM (2014). Nuclear ubiquitination by FBXL5 modulates Snail1 DNA binding and stability. Nucleic Acids Res.

[42] Liu T, Yu J, Deng M, Yin Y, Zhang H, Luo K, Qin B, Li Y, Wu C, Ren T, Han Y, Yin P, Kim J (2017). CDK4/6-dependent activation of DUB3 regulates cancer metastasis through SNAIL1. Nat Commun.

[43] Marotta LL, Almendro V, Marusyk A, Shipitsin M, Schemme J, Walker SR, Bloushtain-Qimron N, Kim JJ, Choudhury SA, Maruyama R, Wu Z, Gönen M, Mulvey LA (2011). The JAK2/STAT3 signaling pathway is required for growth of CD44^+^ CD24^-^ stem cell-like breast cancer cells in human tumors. J Clin Invest.

[44] Su YW, Xie TX, Sano D, Myers JN (2011). IL-6 stabilizes Twist and enhances tumor cell motility in head and neck cancer cells through activation of casein kinase 2. PLoS One.

[45] Joyce JA, Pollard JW (2009). Microenvironmental regulation of metastasis. Nat Rev Cancer.

[46] Zhu Y, Pless M, Inhorn R, Mathey-Prevot B, D’Andrea AD (1996). The murine DUB-1 gene is specifically induced by the betac subunit of interleukin-3 receptor. Mol Cell Biol.

[47] Wu Y, Evers BM, Zhou BP (2009). Small C-terminal domain phosphatase enhances snail activity through dephosphorylation. J Biol Chem.

[48] Dontu G, Abdallah WM, Foley JM, Jackson KW, Clarke MF, Kawamura MJ, Wicha MS (2003). *In vitro* propagation and transcriptional profiling of human mammary stem/progenitor cells. Genes Dev.

[49] Wu Y, Deng J, Rychahou PG, Qiu S, Evers BM, Zhou BP (2009). Stabilization of snail by NF-kappaB is required for inflammation- induced cell migration and invasion. Cancer Cell.

